# Exploring G Protein-Coupled Receptors (GPCRs) Ligand Space via Cheminformatics Approaches: Impact on Rational Drug Design

**DOI:** 10.3389/fphar.2018.00128

**Published:** 2018-03-09

**Authors:** Shaherin Basith, Minghua Cui, Stephani J. Y. Macalino, Jongmi Park, Nina A. B. Clavio, Soosung Kang, Sun Choi

**Affiliations:** College of Pharmacy and Graduate School of Pharmaceutical Sciences, Ewha Womans University, Seoul, South Korea

**Keywords:** GPCR, cheminformatics, drug discovery, ligand-based drug design, structure-based drug design

## Abstract

The primary goal of rational drug discovery is the identification of selective ligands which act on single or multiple drug targets to achieve the desired clinical outcome through the exploration of total chemical space. To identify such desired compounds, computational approaches are necessary in predicting their drug-like properties. G Protein-Coupled Receptors (GPCRs) represent one of the largest and most important integral membrane protein families. These receptors serve as increasingly attractive drug targets due to their relevance in the treatment of various diseases, such as inflammatory disorders, metabolic imbalances, cardiac disorders, cancer, monogenic disorders, etc. In the last decade, multitudes of three-dimensional (3D) structures were solved for diverse GPCRs, thus referring to this period as the “golden age for GPCR structural biology.” Moreover, accumulation of data about the chemical properties of GPCR ligands has garnered much interest toward the exploration of GPCR chemical space. Due to the steady increase in the structural, ligand, and functional data of GPCRs, several cheminformatics approaches have been implemented in its drug discovery pipeline. In this review, we mainly focus on the cheminformatics-based paradigms in GPCR drug discovery. We provide a comprehensive view on the ligand– and structure-based cheminformatics approaches which are best illustrated *via* GPCR case studies. Furthermore, an appropriate combination of ligand-based knowledge with structure-based ones, i.e., integrated approach, which is emerging as a promising strategy for cheminformatics-based GPCR drug design is also discussed.

## Introduction

Rational drug design is the inventive process of identifying pharmaceutically-relevant drug candidates based on the information garnered from a biological target (Jazayeri et al., 2015). Discovery of ligands that modulate a target's activity has contributed largely to the understanding of both physiological and pathological processes (Wacker et al., 2017a). Navigating vast chemical space to identify such ligands seems a daunting task (Oprea and Gottfries, 2001; Lipinski and Hopkins, 2004). Techniques including medicinal chemistry, combinatorial chemistry, and high-throughput screening (HTS) are helpful in the identification of ligands, which can serve as effective modulators for pharmaceutically attractive targets. However, considering the astronomical number of possible drug-like candidates (~10^23^-10^60^), chemical space assessed by experimental techniques is still limited (Rodríguez et al., 2016; Mullard, 2017). In such a scenario, cheminformatics, which belongs to a part of the *in silico* realm, dominates in the exploration of a larger fraction of the chemical space.

Cheminformatics was defined by Brown (1998) as the combination of all available information that can be used in the optimization of a ligand to a potential drug candidate (Bajorath, 2004). This method aids in storing, searching, managing, and analyzing huge amount of chemical data, thereby expediting the development of novel ligand phenotypes (Bajorath, 2004; Valerio and Choudhuri, 2012). Additionally, the extraction of information and knowledge from chemical data could be helpful in the modeling of relationships between chemical structures and biological activities, and in the bioactivity prediction of other compounds from their structures (Schuffenhauer et al., 2006; Humbeck and Koch, 2017). Interestingly, cheminformatics fuses both chemical and biological data from drug candidates and drug targets, respectively, for the identification of new chemical entities (NCEs) and improvement of the reliability of data outcomes.

**Figure 1:**
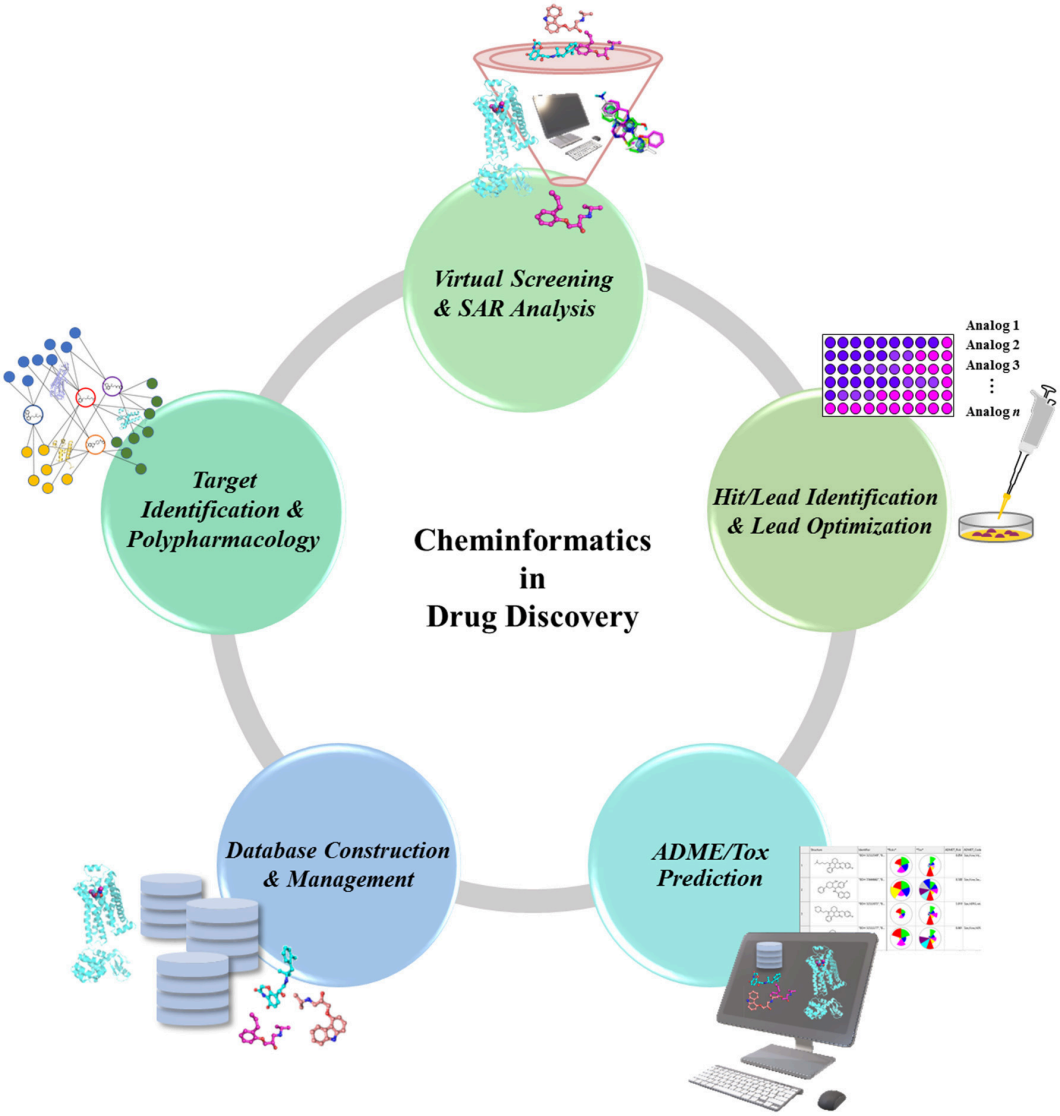
Role of Cheminformatics in the drug discovery process. Cheminformatics is involved in almost every step of the drug discovery pipeline due to the employment and analysis of available data to translate into valuable knowledge, which can in turn be used as a data for further studies.

In the drug discovery pipeline, several cheminformatics approaches play a potent role in the identification of drug target and lead compounds, as well as in the prediction of ADMET properties (Figure 1). Chemogenomics-based databases, as well as computational polypharmacological analyses, have increased in popularity over the last several years as a supplementary method in the identification and validation of potential drug targets (Xie et al., 2014). Once a drug target is identified, the lead candidates with desirable properties are screened out of huge chemical compound libraries, thus underscoring the importance of cheminformatics tools in virtual screening (VS) (Varnek and Tropsha, 2008). Another potent cheminformatics approach, machine-learning is employed for the identification of novel drug candidates from lead compounds *via* generation of computational models (Lee et al., 2010, 2017; Varnek and Baskin, 2012; Mitchell, 2014). Other cheminformatics approaches including similarity and substructure searching could be utilized for the identification of novel scaffolds from large compounds repositories (Vass et al., 2016). The candidate compounds retrieved could be further docked onto the target protein to propose their possible binding affinities toward the target (Lenselink et al., 2016b). Upon identification of the drug-like candidates, these could be further evaluated for ADMET properties using computational models, thus helping in the elimination of undesired compounds at an early stage of drug discovery, and minimizing the time and costs involved.

**Figure 2:**
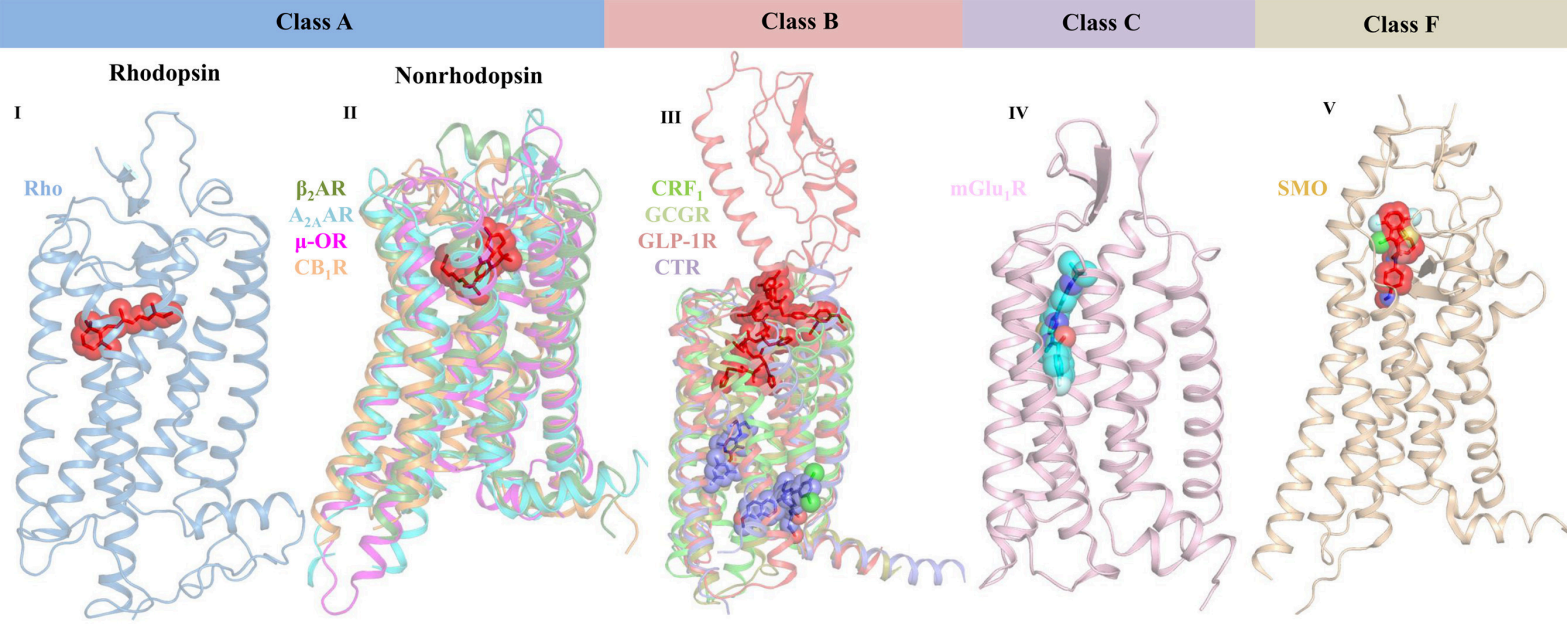
Crystal structures of representative GPCR-ligand complexes from classes A, B, C, and F presenting diverse ligand-binding sites. Class A GPCRs are classified into rhodopsin (bRho, PDB ID: 2HPY) and nonrhodopsin GPCRs. The representative structures of class A nonrhodopsin GPCRs which are further subdivided into aminergic-like (β_2_AR, PDB ID: 3P0G), nucleotide-like (A_2A_AR, PDB ID: 3QAK), peptide-like (μ-OR, PDB ID: 5C1M), and lipid-like receptors (CB_1_R, PDB ID: 5XRA) along with their bound ligands are shown. Similarly, representative structures for class B (CRF_1_ [PDB ID: 4K5Y], GCGR [PDB ID: 5EE7], full-length GLP-1R [PDB ID: 5NX2], and CTR [5UZ7]), class C (mGlu_1_R [PDB ID: 4OR2]), and class F (SMO [PDB ID: 4QIN] bound to negative allosteric modulator) are shown. Receptors are shown in cartoon representation and the ligands are shown as stick models with transparent surfaces. Agonists are represented as red sticks, antagonists are shown as purple sticks, and negative allosteric modulator is shown as blue stick model.

G protein-coupled receptors (GPCRs) belong to a large family of signaling proteins that mediate cellular responses to most hormones, metabolites, cytokines, and neurotransmitters, and therefore serve as “fruitful targets” for drug discovery (Shoichet and Kobilka, 2012). More than 800 genes comprise this receptor family, which modulate several signaling processes involved in behavior, blood pressure regulation, cognition, immune response, mood, smell, and taste (Thomsen et al., 2005). GPCRs are categorized into six classes based on sequence and function, namely Class A—rhodopsin-like receptors, Class B—secretin family, Class C—metabotropic glutamate receptors, Class D—fungal mating pheromone receptors, Class E—cAMP receptors, and Class F—frizzled (FZD) and smoothened (SMO) receptors (Lee et al., 2018). All GPCR members share a common seven transmembrane (7TM) architecture linked by three extracellular (ECL) and three intracellular (ICL) loops (Ciancetta et al., 2015). However, they have low sequence identity and possess different extracellular N-terminal domains and diverse ligand-binding pockets (Figure 2). In case of class A GPCRs, the endogenous ligand is recognized by a ligand-binding site in the 7TM region. For class B GPCRs, the ligand is recognized by both extracellular and 7TM domains. For class C GPCRs, the ligand-binding pocket is found in the extracellular domain (ECD) that contains a Venus flytrap (VFT) module. In case of class F GPCRs, both SMO and FZD receptors possess an ECD that is comprised of an extracellular cysteine-rich domain (CRD) and an ECD linker domain. The endogenous lipoglycoprotein ligand, Wnt binds to the CRD of the FZD receptors (Wang et al., 2013; Wu et al., 2014). Upon ligand binding, GPCRs activate at least one of the two signaling partners, namely heterotrimeric GTP-binding proteins (G-proteins) or β-arrestins, and mediate signal flow *via* modulation of various downstream effectors.

GPCR drug discovery has been successful and many of the world's top-selling drugs have targeted this receptor family (Sriram and Insel, 2018). Class A GPCRs are the most immensely investigated GPCR drug target within the drug market due to their centrality in diseases, structural availability, and relative ease of accessibility. The high druggability of GPCRs and its central role in diseases (including alzheimer's disease, cancer, diabetes, obesity, and psychiatric disorders) provide a strong spearhead for its continuous efforts in drug discovery and development (Tautermann, 2016). A recent study of all GPCR drugs and agents currently in clinical trials revealed that 475 drugs (i.e., ~34% of all drugs approved by Food and Drug administration [FDA]) mediate their effects through 108 unique GPCRs (Hauser et al., 2017). Additionally, the success rates for GPCR-targeted agents in the last 5 years were 78% (phase I), 39% (phase II), and 29% (phase III) (Hauser et al., 2017). The most recently FDA approved GPCR-targeted drug is Zilretta (triamcinolone acetonide extended-release injectable suspension), a glucocorticoid receptor agonist, which is used for the pain management of knee osteoarthritis (https://www.drugs.com/history/zilretta.html).

To utilize cheminformatics approaches in GPCR drug design, understanding the nature of the ligands, structural intricacies of the receptor, ligand-receptor interactions, and interaction of the receptors with downstream signaling complexes or other signaling partners is essential. Additionally, unveiling the relationships among ligand, receptor, and effector is necessary to investigate positive and negative allosterism, inverse agonism, biased signaling, and multimeric receptor pharmacology (Lane et al., 2017). Recent upsurge in the crystal structures of GPCRs provides a robust, 3D structural framework for identification of pharmaceutically-relevant ligands using ligand– and structure-based computational approaches, including molecular modeling of receptor dynamics, ligand docking, and virtual ligand screening (VLS) (Coudrat et al., 2017a). Following the successful application of VLS approaches in targets such as kinases, proteases, and other enzyme families, it is also becoming a popular ligand screening tool for GPCRs (Heifetz, 2018). The success of structure-based VLS could be visualized by the encouragingly high hit-rates ranging from 20 to 70% in the identification of novel ligands for several class A GPCRs (Table 1).

**Table 1:** Key details of GPCR virtual screening campaigns reported in the last 5 years (2013–2017).

**GPCR class and classification type**	**Receptor type**	**VS library and size**	**Hits/hit rate**	**Structure of notable hits**	**References**
A, nonrhodopsin (aminergic)	β_2_AR	ZINC database: (a) 2.7 million lead-like subset (b) 400k fragment-like subset	6 hits (27.3%)	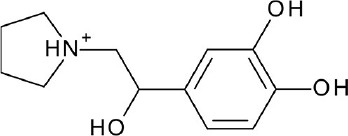	Weiss et al., 2013
				pKi = 3.9	
A, nonrhodopsin (aminergic)	D_2_R	ZINC database: (a) 2.7 million lead-like subset (b) 400k fragment-like subset	3 hits (20%)	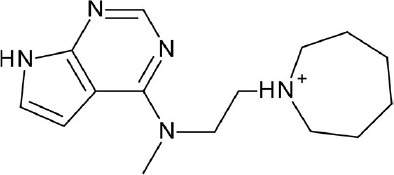	Weiss et al., 2013
				pEC_50_ = 4	
A, nonrhodopsin (aminergic)	M_2_R	ZINC database: 3.1 million compounds	11 of 19 (57.9%)	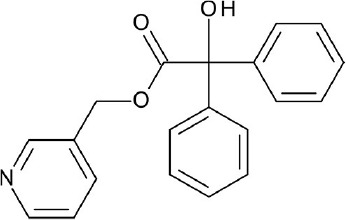	Kruse et al., 2013
				Ki = 1.2 uM	
A, nonrhodopsin (aminergic)	M_3_R	ZINC database: 3.1 million compounds	8 of 16 (50%)	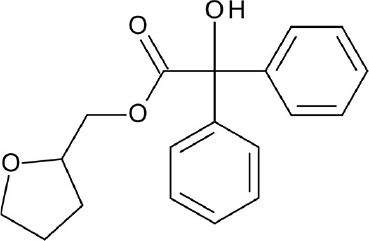	Kruse et al., 2013
				Ki = 1.2 uM	
A, nonrhodopsin (lipid-like)	CB_2_R	Enamine, Otava, ChemBridge, ChemDiv, Vitasm, IBS, LifeChemicals, Specs, and TimTec: 5,613,820 compounds	13 hits ≥ 50% inhibition at 10 uM (13.4%)	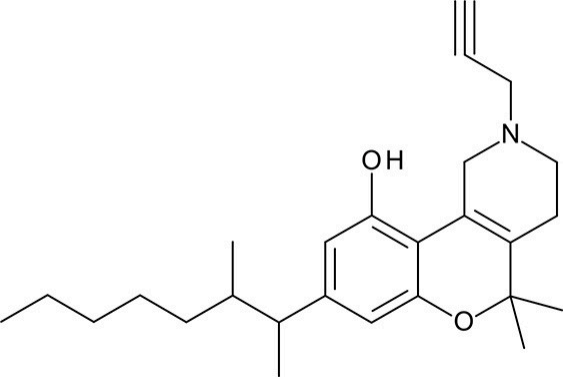	Renault et al., 2013
				Ki = 2.3 nM	
A, nonrhodopsin (aminergic)	AgOAR45B	ZINC drug-like subset: 12 million compounds	45 hits (64.3%)	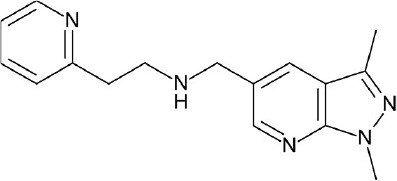	Kastner et al., 2014
				Ki = 2.7 uM	
A, nonrhodopsin (aminergic)	5-HT_1A_R	WDI, PCL, TimTec, and ASINEX: 80,800 compounds	9 hits ≥ 50% inhibition at 10 uM (60%)	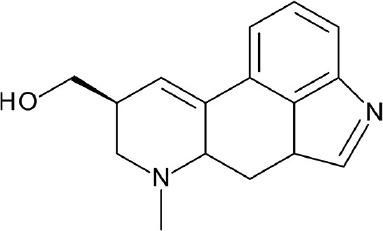	Luo et al., 2014
				IC_50_ = 2.3 nM	
A, nonrhodopsin (peptide-like)	NOP receptor	ZINC database CNS Permeable subset: 400,000 compounds	6 hits ≥ 50% inhibition at 300 uM (30%)	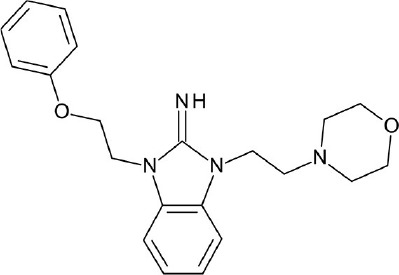	Daga et al., 2014
				K_i_ = 1.42	
A, nonrhodopsin (peptide-like)	PAR2	FDA-approved drugs: 1,216 compounds	4 hits ≥ 50% inhibition at 30 uM	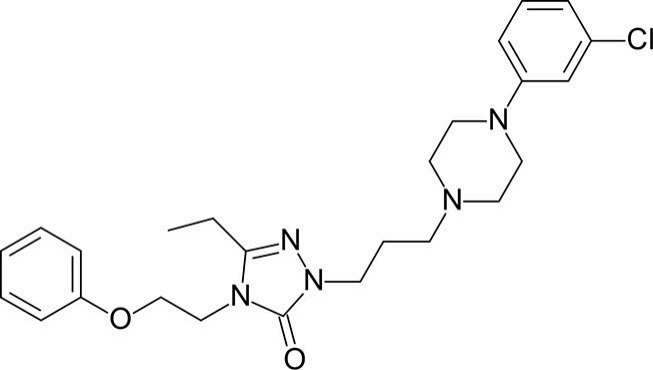	Xu et al., 2015
				IC_50_ = 10 uM	
A, nonrhodopsin (aminergic)	5-HT_6_R	ChEMBL: 12,608 compounds	6 hits (16.7%)	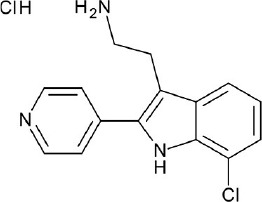	Kelemen et al., 2016
				IC_50_ = 0.1 uM	
A, nonrhodopsin (aminergic)	H_1_R	ChEMBL: 108,790 compounds	19 hits (73.1%)	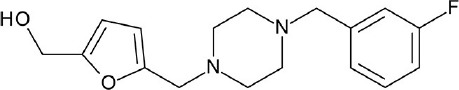	Kooistra et al., 2016
				pK_i_ = 4.72	
A, nonrhodopsin (aminergic)	β_2_AR	ChEMBL: 108,790 compounds	18 hits (52.9%)	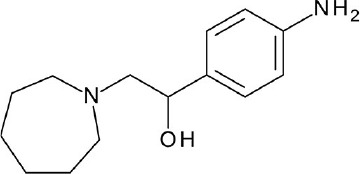	Kooistra et al., 2016
				pEC_50_ = 4.52	
C, metabotropic glutamate	mGlu_1_R	Asinex: 695,855 compounds	5 hits (14.3%)	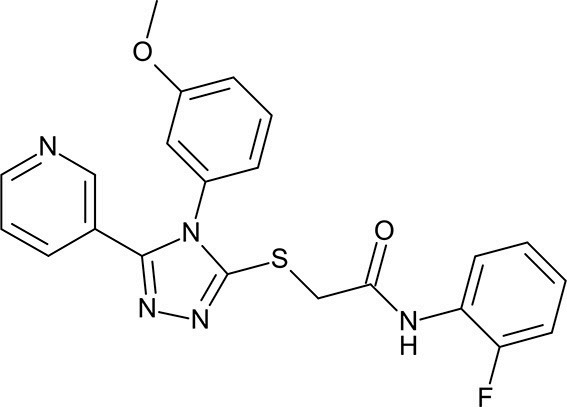	Jang et al., 2016
				IC_50_ = 10.22 uM	
	FGSG_02655 (Class I, pheromone receptor)	Life Chemicals GPCR Targeted Libraries: 11,571 compounds	10 VS hits	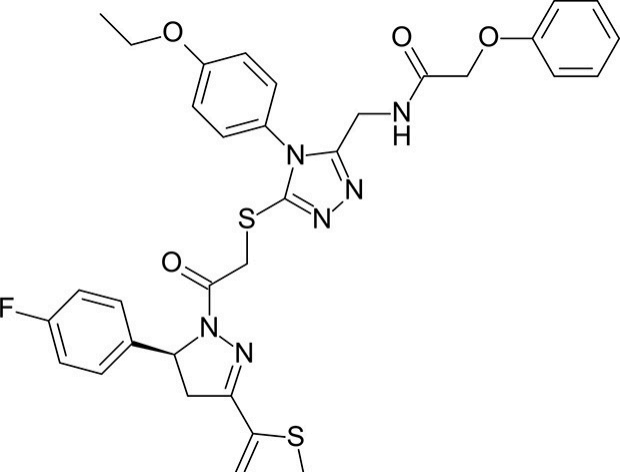	Bresso et al., 2016
A, nonrhodopsin (peptide-like)	PAR2	(a) Asinex: 433,973 compounds (b) ChemDiv: 1,213,470 compounds	3 hits ≥ 30% inhibition at 10 uM (6.4%)	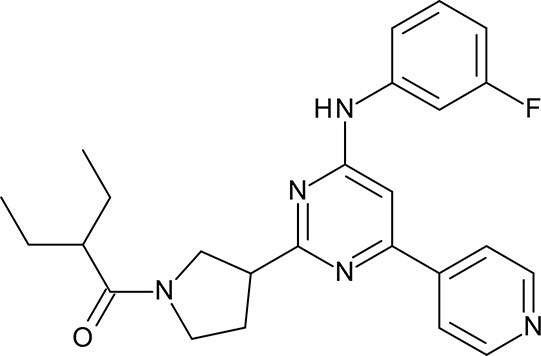	Cho et al., 2016
				IC_50_ = 8.22 uM	
A, nonrhodopsin (peptide-like)	NTSR1	ZINC, ChemBridge, and J&K: 1,000,000 compounds	4 hits (9.1%)	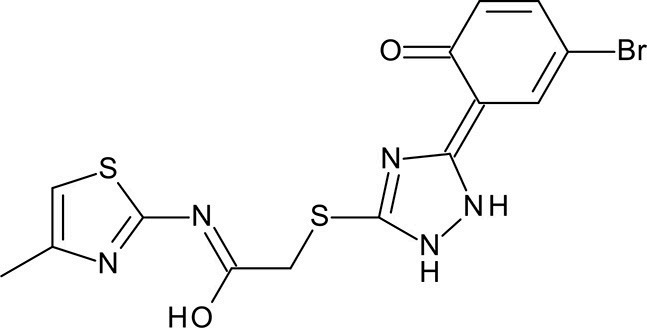	Zhang et al., 2016
				IC_50_ = 14.47 uM	
A, nonrhodopsin (aminergic)	5-HT_2A_R	ZINC Clean Lead-like subset: 140,809 compounds	15 VS hits	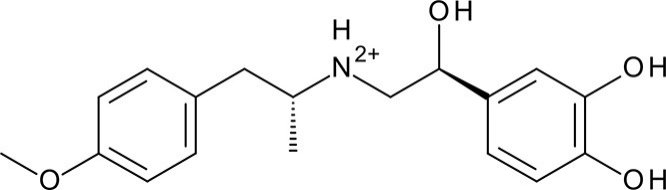	Gandhimathi and Sowdhamini, 2016
A, nonrhodopsin (aminergic)	D_2_R	6,500,000 compounds	10 hits (47.6%)	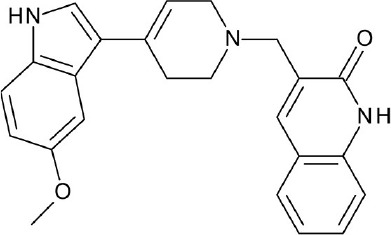	Kaczor et al., 2016
				Ki = 58.1	
A, nonrhodopsin (aminergic)	M_2_R	NCI Diversity Set: 1,600 compounds	19 hits (50%)	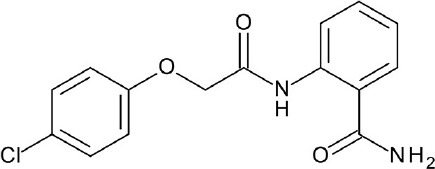	Miao et al., 2016
				pKi = 3.8	
A, nonrhodopsin (aminergic)	H_3_R	Phase database	6 hits (8%)	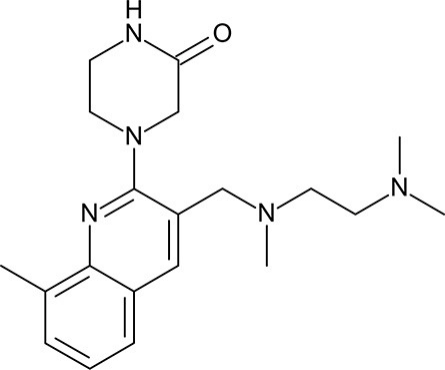	Frandsen et al., 2017
				pKi = 6.1	
A, rhodopsin	GPR91	ZINC In-Stock subset: 12,782,590 compounds	12 hits (10.8%)	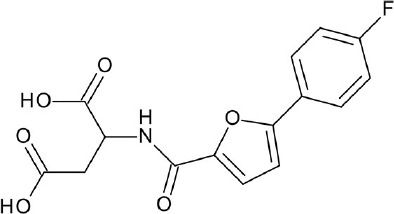	Trauelsen et al., 2017
				EC_50_ = 1.9 uM	

In this review, we deliver a comprehensive assessment on the state-of-the-art cheminformatics approaches for GPCR drug discovery with successful models from literature. Firstly, insights on GPCR ligand space and its recent structural advances are summarized. Subsequently, the key principles and boundaries of ligand–, structure-based, and integrated cheminformatics approaches in GPCR drug discovery are discussed in the main text. We also shed some light on the contemporary cheminformatics tools utilized in GPCR drug discovery. Additionally, the limitations associated with cheminformatics approaches have been discussed, which could assist the reader to rationale the best *in silico* tool during their research. Lastly, we conclude with a summary of the review contents and prospects of the cheminformatics approaches in GPCR drug discovery.

## Booming age of GPCR structural biology

The pioneering study of two-dimensional (2D) structure for bovine rhodopsin (bRho) in 1983 marked the beginning of GPCR structural biology (Hargrave et al., 1983). A decade later, 2D projection map was calculated from the solved 2D crystals of bRho using electron cryomicroscopy, which served as the basis for the construction of the receptor molecular model (Baldwin, 1993; Schertler et al., 1993). However, the first three-dimensional (3D) structure of bRho in its inactive state was released only in 2000 (Palczewski et al., 2000). Despite relentless efforts, elucidation of GPCR structures remained challenging due to several factors, including maintenance of structural integrity of the receptors by embedding in a membrane-like environment, presence of flexible ECLs and ICLs, low expression level of the receptor, and displaying basal signaling activity even in the absence of a ligand. However, all the aforementioned problems have been circumvented with the advances in GPCR crystallography, protein engineering, and innovations in biotechnology. Introduction of small, stable fusion proteins (T4 lysozyme and b562RIL) decreased the flexibility of the receptor regions (ICL3, ICL2, and N-terminal regions), and improved the crystal contacts. Likewise, antibody fragments or nanobodies improved the conformational stability of the receptors. Insertion of mutations (stabilized receptor (StaR) approach) enhanced the receptor thermostability in a particular conformational state and increased the protein expression levels.

The first structural breakthrough of a human GPCR, i.e., β_2_-adrenergic receptor (β_2_AR with a diffusible ligand), using different crystallization techniques came in 2007 (Cherezov et al., 2007). Moreover, the first crystal structures for GPCR classes B, C, and F have been solved (Hollenstein et al., 2013; Wang et al., 2013; Wu et al., 2014). So far, experimental structures of 44 distinct GPCRs and ~205 ligand-receptor complexes covering all the four classes, A–C, and F are available, of which most belong to the Class A subfamily (Hauser et al., 2017). It is to be noted that most of the existing GPCR structures are inactive ones, bound to an inhibitor. In the last year (2017) alone, more than 40 GPCR crystal structures have been determined which are listed in Table 2. GPCR structural studies have revealed the arrangement of the TM domains, location of the orthosteric, allosteric, bitopic, and biased ligand binding sites, homo– or heterooligomerization of receptors, and structural rearrangements involved in conformational changes upon GPCR activation or inactivation (Manglik and Kruse, 2017; Schrage and Kostenis, 2017). Besides garnering these 3D structural insights, the molecular basis of GPCR signal transduction coupled to G-proteins or β-arrestins were elucidated through X-ray crystallography and electron cryomicroscopy techniques. Oligomeric complex structures of bRho coupled to G-protein peptide (Rho/GαCT) (Scheerer et al., 2008), human Rho coupled to visual arrestins (Kang et al., 2015; Zhou et al., 2017), β_2_AR coupled to Gs-protein (Rasmussen et al., 2011) and β-arrestin 1 (Shukla et al., 2014), A_2A_ adenosine receptor (A_2A_AR) in complex with a mini-G_s_ protein (Carpenter et al., 2016), glucagon-like peptide 1 receptor (GLP-1R) in complex with a Gs-protein (Zhang et al., 2017), and calcitonin receptor (CTR) coupled to Gs-protein (Liang et al., 2017) have been elucidated. These complex structures provide full mechanistic insights into GPCR and biased signaling, thus underpinning their functional significance and pharmacological targeting.

**Table 2:** Summary of GPCR solved structures released in the past 1 year (Dec ‘16-Nov ‘17).

**Class type, classification**	**Receptor type**	**Species**	**Ligand**	**Ligand type**	**Released year**	**Resolution**	**PDB ID**
A, rhodopsin	Rhodopsin^a^	Human	N/A	N/A	2017	3.0	5W0P
	Rhodopsin^b^	Bovine	N/A	N/A	2017	2.7	5TE3
	Rhodopsin	Bovine	10,20-Methanoretinal	Agonist	2017	4.0	5TE5
A, nonrhodopsin (aminergic receptors)	β_2_AR	Human	Carazolol; 4-carbamoyl-N-[(2R)-2-cyclohexyl-2-phenylacetyl]-L-phenylalanyl-3-bromo-N-methyl-L-phenylalaninamide	Inverse agonist; Allosteric antagonist	2017	2.7	5X7D
	D_4_R	Human	Nemonapride	Antagonist	2017	2.0	5WIU
	D_4_R	Human	Nemonapride	Antagonist	2017	2.1	5WIV
	5-HT_2B_	Human	Lysergic acid diethylamide	Agonist	2017	2.9	5TVN
	5-HT_2B_	Human	Ergotamine	Agonist	2017	3.0	5TUD
A, nonrhodopsin (nucleotide-like receptors)	A_1_AR	Human	DU172	Covalent antagonist	2017	3.2	5UEN
	A_1_AR	Human	PSB36	Antagonist	2017	3.3	5N2S
	A_2A_AR	Human	ZM241385	Inverse agonist	2017	1.7	5NM4
	A_2A_AR	Human	ZM241385	Inverse agonist	2017	2.0	5NM2
	A_2A_AR	Human	ZM241385	Inverse agonist	2017	2.1	5NLX
	A_2A_AR	Human	Theophylline	Antagonist	2017	2.0	5MZJ
	A_2A_AR	Human	PSB36	Antagonist	2017	2.8	5N2R
	A_2A_AR	Human	Caffeine	Neutral antagonist	2017	2.1	5MZP
	A_2A_AR	Human	ZM241385	Inverse agonist	2017	2.8	5JTB
	A_2A_AR	Human	ZM241385	Inverse agonist	2017	3.2	5UVI
	A_2A_AR	Human	5-Amino-N-[(2-Methoxyphenyl)methyl]-2-(3-Methylphenyl)-2h-1,2,3-Triazole-4-Carboximidamide	Bitopic antagonist	2017	3.5	5UIG
A, nonrhodopsin (peptide-like receptors)	CCR2	Human	BMS-681; CCR2-RA-[R]	Orthosteric antagonist; Allosteric antagonist	2016	2.8	5T1A
	CCR5	Human	5P7-CCL5	Antagonist	2017	2.2	5UIW
	CCR9	Human	Vercirnon	Allosteric antagonist	2016	2.8	5LWE
	NTSR1	Rat	NTS_8−13_	Agonist	2016	3.3	5T04
	APJR	Human	AMG3054	Agonist	2017	2.6	5VBL
	PAR2	Human	AZ3451	Allosteric antagonist	2017	3.6	5NDZ
	PAR2	Human	AZ8838	Antagonist	2017	2.8	5NDD
	PAR2	Human	AZ7188	Antagonist	2017	4.0	5NJ6
	AT_2_R	Human	N-benzyl-N-(2-ethyl-4-oxo-3-{[2′-(2H-tetrazol-5-yl)[1,1′-biphenyl]-4-yl] methyl}-3,4-dihydroquinazolin-6-yl)thiophene-2-carboxamide	Antagonist	2017	2.8	5UNG
	AT_2_R	Human	N-[(furan-2-yl)methyl]-N-(4-oxo-2-propyl-3-{[2′-(2H-tetrazol-5-yl)[1,1′- biphenyl]-4-yl]methyl}-3,4-dihydroquinazolin-6-yl)benzamide	Dual antagonist	2017	2.9	5UNH
	AT_2_R	Human	N-benzyl-N-(2-ethyl-4-oxo-3-{[2′-(2H-tetrazol-5-yl)[1,1′-biphenyl]-4-yl]	Antagonist	2017	2.8	5UNF
	ET_B_R	Human	Bosentan	Dual antagonist	2017	3.6	5XPR
	ET_B_R	Human	K-8794	Antagonist	2017	2.2	5X93
A, nonrhodopsin (lipid-like receptors)	FFAR1	Human	MK-8666; AP8	Partial agonist; Full allosteric agonist	2017	3.2	5TZY
	FFAR1	Human	MK-8666	Partial agonist	2017	2.2	5TZR
	LPA_6_R^b^	Zebrafish	N/A	N/A	2017	3.2	5XSZ
	CB_1_R	Human	AM11542	Full agonist	2017	2.8	5XRA
	CB_1_R	Human	AM841	Full agonist	2017	3.0	5XR8
	CB_1_R	Human	Taranabant	Inverse Agonist	2016	2.6	5U09
B, secretin-like receptors	GLP-1R	Human	Truncated peptide	Agonist	2017	3.7	5NX2
	GLP-1R	Human	PF-06372222	Negative allosteric modulator	2017	2.7	5VEW
	GLP-1R	Human	NNC0640	Negative allosteric modulator	2017	3.0	5VEX
	GLP-1R^c^	Rabbit	GLP-1	Agonist	2017	4.1	5VAI
	GCGR	Human	NNC0640	Negative allosteric modulator	2017	3.0	5XEZ
	GCGR	Human	NNC0640	Negative allosteric modulator	2017	3.2	5XF1
	CTR^c^	Human	sCT	Agonist	2017	4.1	5UZ7

a *Arrestin-bound state of the receptor*.

b *Ligand-free basal state of the receptor*.

c *Fully-active receptor complexed with a G protein*.

GPCRs are known to exist or function as monomers, dimers, and/or higher order oligomers, including homo– or hetero– dimers/oligomers (Guo et al., 2017). In addition to the accumulated experimental data through biochemical and biophysical techniques, the structural information on GPCR dimers or higher order oligomers were provided by X-ray crystallography. The first reported higher-order crystal structure of Rho and opsin in native membranes were reported in 2003 (Liang et al., 2003). Consequently, several structures including rhodopsin and nonrhodopsin class A GPCRs were elucidated (Lee et al., 2018). The oligomeric structures of GPCRs are essential for modulation of receptor function, mediation of cross-talk between GPCRs or other signaling pathways, and cellular trafficking, hence they have been associated with specific functional effects. Moreover, targeting these oligomeric structures as drug candidates could provide a new arena for drug development and specificity. The wealth of information supporting the existence of homo- and heterooligomers of GPCRs can be retrieved from the RCSB PDB (https://www.rcsb.org/pdb/home/home.do) or GPCR Oligomerization Knowledge Base (Khelashvili et al., 2010). In addition to these structural intricacies, GPCR signaling is also modulated by the presence of ligands other than orthosteric, which will be discussed in the following sections. Furthermore, adding details like GPCR dynamics to the structural information would provide a bigger picture to the biomedical researchers in this field. Such dynamic events triggered upon receptor activation or inhibition mechanisms could be covered by powerful methodologies including, bottom-up Hydrogen Deuterium eXchange Mass Spectrometry (HDX-MS) and resonance energy transfer (RET) (Li et al., 2015; Zhang, 2017). These important structural tools aid in better GPCR drug design by adding valuable information to our understanding of GPCR function, dynamics, protein-protein interactions, and receptor-ligand interactions (Vilardaga, 2011; Kauk and Hoffmann, 2017). Collectively, all the structural studies provide unprecedented insights into the structural and functional diversity of this receptor family. The wealth of structural information on all GPCRs is invaluable for ligand-based drug design (LBDD), structure-based drug design (SBDD), and integrated paradigms which complement traditional drug discovery efforts.

## Insights into GPCR ligand space

Various signaling pathways involve several GPCRs whose activities are mediated by ligand binding. Based on activation intensity, GPCR modulators can be divided into agonists, partial agonists, antagonists, and inverse agonists. Full agonists can stimulate maximal GPCR activity leading to recruitment of downstream proteins for signal transduction. Partial agonists, on the other hand, cannot induce 100% activation of receptors and acts as a type of antagonist while in the presence of full agonists. However, it can act as full agonists when there are excess receptors and in the absence of actual full agonists. Antagonists act as agonist blockers and can be divided into neutral antagonists and inverse agonists. Neutral antagonists can bind to GPCRs but do not affect the receptor's constitutive activity, whereas inverse agonists can block agonist effects. These modulators can directly interact with the orthosteric binding site of GPCRs (Wacker et al., 2017a).

While structural architecture of the TM region is largely conserved, the remarkable diversity in GPCR sequences are most notable in the ECL and ICL regions. This leads credence to the capacity of the GPCR family to interact with a wide range of ligands that vary in size, shape, and physicochemical properties, most of which bind to the orthosteric site to modulate receptor activity. In the ECL region, ECL2 plays a critical role in ligand recognition, access, and selectivity (Dror et al., 2011; Kruse et al., 2012; Zhang H. T. et al., 2015). For Class A GPCRs, lipophilic ligands often come from the lipid membrane and access the orthosteric site through the “lid” formed by the N-terminus and ECL2. In the case of hydrophilic ligands, ECL2 of different receptors only partially covers the ECL region through a variety of structures that shapes the entrance to the binding pocket (Venkatakrishnan et al., 2013). On the other hand, modulators of class B GPCRs are frequently peptide ligands, which possess large volume and high flexibility, requiring a more solvent-accessible orthosteric binding pocket (Liang et al., 2017).

**Figure 3:**
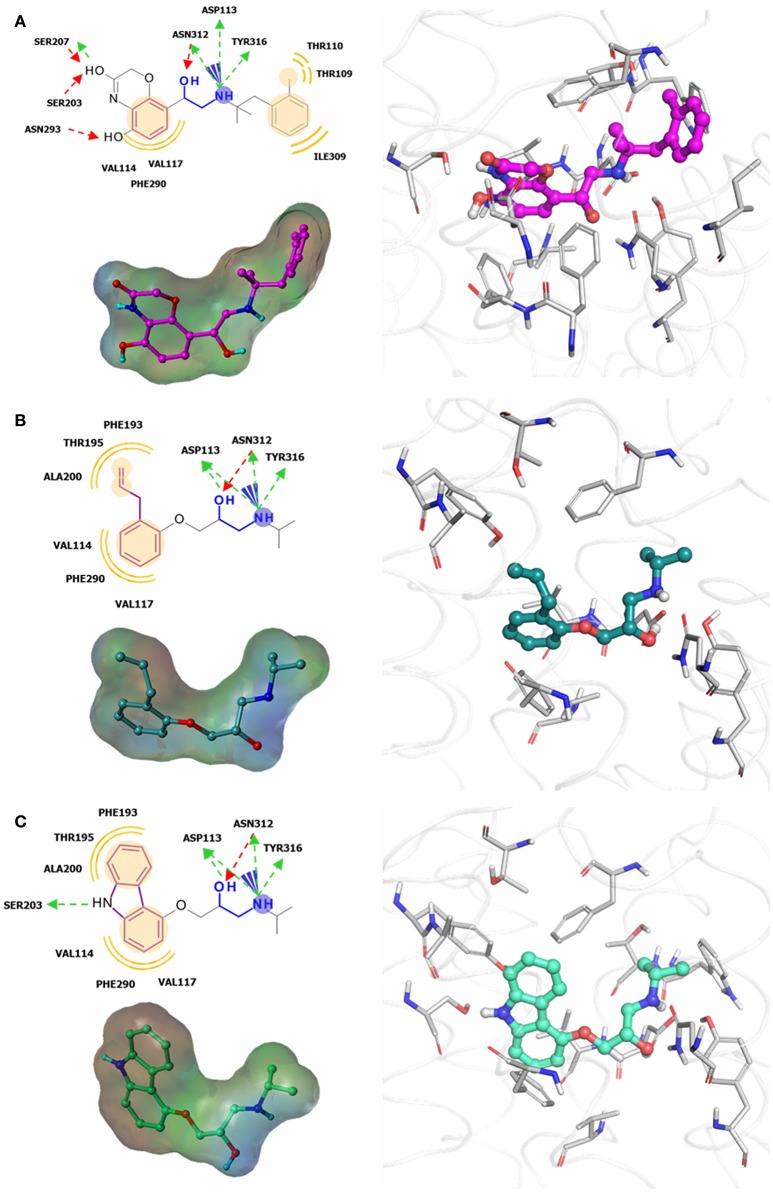
Examples of β2-adrenergic receptor (β2AR) orthosteric ligands with similar structures but possess different activities. **(A)** BI167,107 acts an agonist (PDB ID: 4LDE) (Ring et al., 2013), **(B)** alprenolol acts an antagonist (PDB ID: 3NYA) (Wacker et al., 2010), and **(C)** carazolol acts as an inverse agonist (PDB ID: 2RH1) (Cherezov et al., 2007).

Increase in static GPCR structures and advances in MD facilities have assisted in the elucidation of GPCR-ligand binding interaction. A thorough investigation of the ligand binding pocket of several GPCRs indicated the presence of multiple topologically equivalent residues that forms a consensus ligand binding network in almost all Class A receptors, providing an explanation for cross-reactivity and polypharmacology. Moreover, deviations from these consensus binding residues can account for ligand specificity in different GPCR members, and can thus be exploited in the design of specific and potent ligands (Venkatakrishnan et al., 2013). Regardless of the upsurge in information in the last few decades, it is still difficult to understand the differences in ligand binding requirements for agonists, antagonists, and inverse agonists of a given receptor, despite having almost identical structures (Figure 3). This calls for more studies focused on identifying key residues for agonism and antagonism, not only ligand binding specificity. Along with this, it is important to scrutinize activity cliffs of ligands as significant shifts in modulation type could be observed through small changes in ligand structures.

Besides the orthosteric site, GPCR ligands can also bind to allosteric pockets and indirectly modulate receptor activity. Allosteric modulators can be divided into two types: (a) positive allosteric modulators (PAMs), which increases agonist affinity, and (b) negative allosteric modulators (NAMs), which acts as an allosteric antagonist or inverse agonist to decrease agonist affinity (Christopher et al., 2013, 2015; Kenakin, 2016). Additionally, there are some molecules that can both interact with orthosteric and allosteric sites, known as bitopic modulators (Dror et al., 2013; Fronik et al., 2017). Allosteric modulators can be either endogenous molecules, like sodium and cholesterol (Katritch et al., 2014), or exogenous molecules like natural products and synthetic compounds. Since allosteric modulators bind to sites other than the orthosteric site, they can co-bind with the putative ligand on the receptor to alter conformation and activity, thus affecting downstream signaling.

In case of CC chemokine receptor type 9 (CCR9), vercirnon (antagonist) was co-crystallized and unexpectedly found to interact with the intracellular binding site, blocking G-protein coupling (Oswald et al., 2016). Another example of an allosteric modulator is 1-(2-(2-(tert-butyl)phenoxy)pyridin-3-yl)-3-(4-(trifluoromethoxy)phenyl)urea (BPTU), which binds outside the purinergic P2Y_1_ receptor, flanking the TM bundle inside the lipid bilayer. While BPTU shows lower potency than known orthosteric antagonist, MRS2500, its allosteric interactions allow higher selectivity for the P2Y_1_ receptor (Zhang D. et al., 2015). Apart from small molecule compounds, ions can also function as an allosteric modulator, as illustrated by the discovery of the conserved allosteric binding pocket for Na^+^ in Class A GPCRs (Katritch et al., 2014).

**Figure 4:**
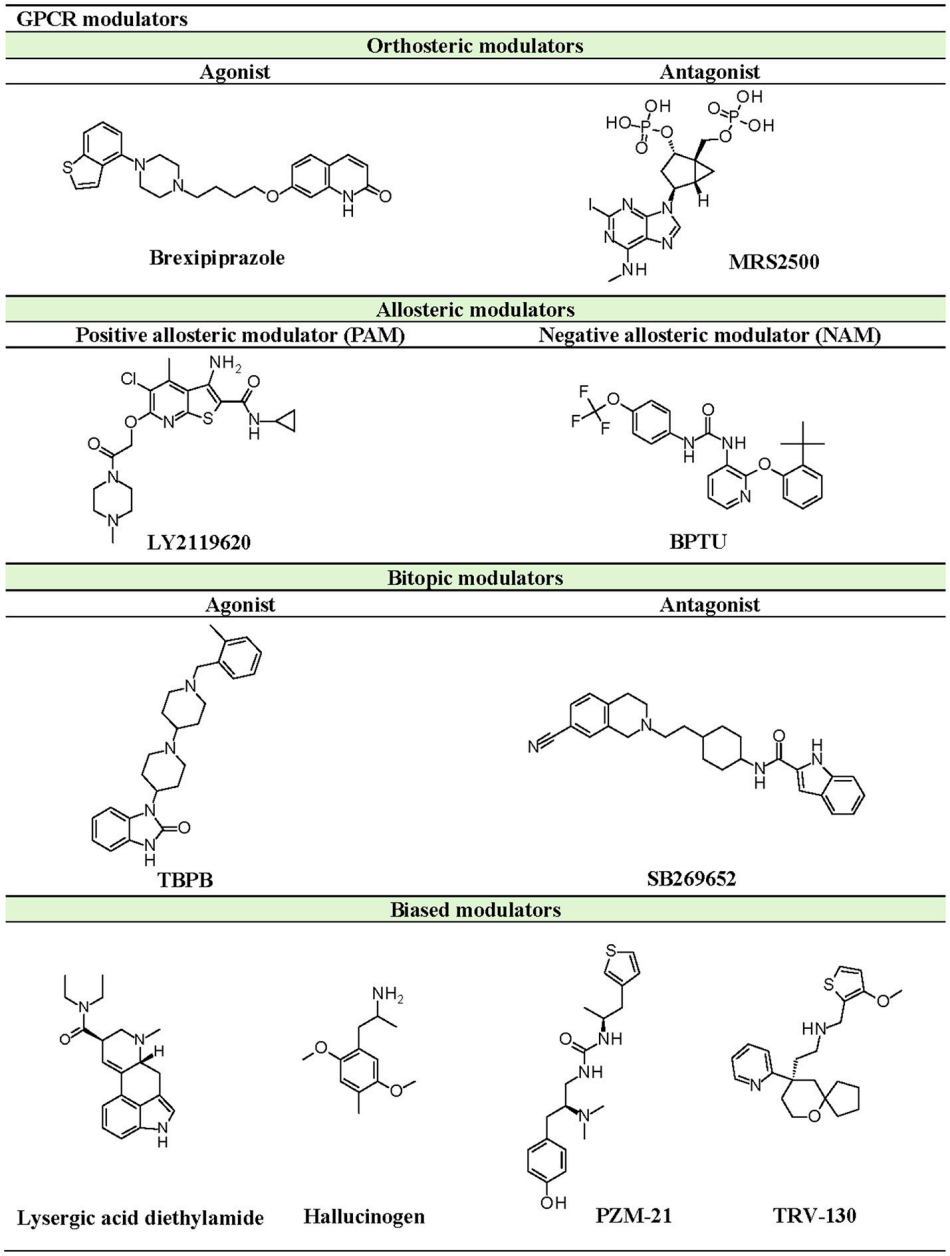
Representative chemical structures of various GPCR modulators.

The current rising star in GPCR research is biased signaling. Previously, GPCRs were presumed to exist as a simple two-state receptor model [“on” (activation) and “off” (inactivation)]. However, extensive analyses of different signaling pathways paved way to an exciting discovery that GPCRs have multiple conformations, each tailored to a specific response and downstream effect. Different ligands induce different receptor conformations, and each conformational state could initiate a specific downstream signal. While this finding increases the difficulty in drug discovery and design, there is also an opportunity to selectively block pathways implicated in various pathologies, while leaving normal homeostatic processes intact (Bologna et al., 2017). Typically, G protein signaling occurs upon agonist binding, whereas arrestin-mediated signaling occurs through arrestin binding. In this instance, GPCR drug design strategy could be dependent on identifying agonists biased for either G protein or arrestin signaling, leading to higher drug efficacy and diminished adverse effects (DeWire and Violin, 2011). Some excellent examples of biased ligands include lysergic acid diethylamide (LSD) (Wacker et al., 2017b), a well-known hallucinogen which appears to display bias toward β-arrestin signaling, and synthetic opioids TRV-130 (DeWire et al., 2013) and PZM-21 (Manglik et al., 2016), which are biased toward G protein signaling. Altogether, these accumulated data may provide extremely beneficial hints in the discovery and design of GPCR ligands based on the intended activity and targeted pathology. Figure 4 depicts some of the common GPCR modulators that are distinguished by activity types.

## Cheminformatics-based paradigms in GPCR drug discovery

### Cheminformatics approaches based on the knowledge derived from GPCR ligands

Cheminformatics tools are frequently utilized in GPCR research due to the enormous amount of GPCR ligand data. Difficulties in crystallizing membrane proteins and receptor flexibility hindered structural elucidation and drug discovery research for this receptor. Due to these shortcomings, ligand-based approaches started to thrive in order to provide a better understanding of GPCR function and pharmacology. Some of the major ligand-based cheminformatics approaches are detailed below.

#### Cheminformatics and virtual screening

*In silico* screening method started to become popularly used after the integration of high throughput screening (HTS) and information technology (Coudrat et al., 2017b). Several computational and VS methods are frequently utilized in different stages of drug discovery and development, but some of the earliest and most commonly used ones are similarity- and QSAR-based strategies due to their efficiency and capability in analyzing simple 2D structures. These strategies are dependent on the principle that similar structures are predicted to display similar activities. Similarity-based methods need at least one established hit whose chemical structure is used to calculate pertinent molecular fingerprints, which is then employed to screen chemical libraries for compounds containing similar structure or fragments. On the other hand, QSAR-based strategies rely on the developed mathematical models which require an adequate number of biologically active compounds with activities covering a wide span of concentration. In this case, screening is dependent on the quality of the dataset used and the accuracy of the developed model (Luo et al., 2016).

Similarity-based VS was applied in a recent study for the discovery of a novel series of cannabinoid receptor 2 (CB_2_R) agonists (Gianella-Borradori et al., 2015). CB_2_R is a class A, lipid-like GPCR that regulates the effects of endogenously produced cannabinoid receptor ligands and has been implicated in several inflammatory diseases. In this study, an in-house database containing around 25,000 compounds was screened based on 40 low-energy conformations of known active and selective compound HU-308. Compounds were ranked based on their similarity with any of the 40 conformers of HU-308, and the top 94 were selected for biochemical screening based on the combined color score, which refers to chemistry alignment akin to pharmacophore features, and shape Tanimoto score, which accounts for 3D conformer overlay. From the initial hits, the top 16 active compounds displayed 6 new core scaffolds. Upon combined inspection of bioactivity, molecular weight, and lipophilicity, DIAS1 was chosen and used for further mining of the in-house library with the help of the newly identified scaffold. The second VS led to the discovery of DIAS2, which exhibited better activity and reduced lipophilicity as compared to DIAS1. Further structure-activity relationship (SAR) studies were performed for the optimization of the lead compound to improve potency, selectivity, and pharmacokinetic properties, resulting in candidate compounds that show nanomolar activity and selectivity for CB_2_R.

Another study used the US EPA's ToxCast database to develop QSAR models for 18 aminergic GPCRs (Mansouri and Judson, 2016). While the ToxCast program can screen hundreds of compounds *in vitro* to determine toxicity, the chemical space covered by their database is not enough to include all compounds of interest. However, the database can be employed in the development of predictive QSAR models. Two QSAR models were developed during the study, a qualitative (active vs. non-active) and a quantitative (potency value prediction) model. Various descriptors were calculated from the 2D structures of the compounds in the database and were subjected to genetic algorithms (GAs) to identify the best and most predictive descriptors. Several model-fitting methods, including PLSDA (partial least square discriminant analysis), SVMs (support vector machines), kNNs (k-nearest neighbors), and PLSs (partial least squares), were used to generate the QSAR models, which were later evaluated for accuracy and predictability. As a result, they were able to produce suitable models for aminergic GPCR assays and demonstrate the reliability of QSAR-based methods for analysis.

#### Cheminformatics and *de novo* ligand design

Typically, ligand-based *de novo* drug design utilizes approved drugs or known inhibitors as reference structures or a source of pharmacophores that are relevant for bioactivity to build new chemical structures. While novelty and potency are always favored in drug discovery research, *de novo* structures should also have desirable pharmacokinetic properties (Kawashita et al., 2015). The combination of *de novo* drug design and computer-aided VS, along with the application of ADME/Tox models for the prediction of pharmacokinetic properties, has the capability of more effectively identifying NCEs with the desirable pharmacological activity profiles. In this sense, *de novo* drug design approach has become the forerunner of the long-envisioned personalized medicine where patients can be given custom-tailored drugs with increased efficacy and reduced adverse effects.

Rodrigues et al. worked on 5-hydroxytryptamine receptor subtype 2B (5-HT_2B_) drug discovery and were able to identify selective ligands through multidimensional *de novo* design (Rodrigues et al., 2015). In the Molecular Ant Algorithm (MAntA) software (Reutlinger et al., 2014), chemically advanced template search version 2 (CATS2), pharmacophores, and Morgan substructure fingerprints were employed to generate 5-HT_2B_ selective ligands via reductive amination, resulting in over 5,000 new compound structures from which 4 were selected based on calculated 5-HT_2B_ selectivity. To further improve selectivity and increase the scaffold diversity, *de novo* design software DOGS (Hartenfeller et al., 2012) and FDA-approved drug molecule structures were utilized to produce NCEs. The resulting compounds were screened with PAINS (Baell and Holloway, 2010) and ADMET filters (Lagorce et al., 2008) to remove undesirable molecules before performing experimental validation assays. Finally, four more compounds were obtained and among them, one compound showed promising selectivity for the 5-HT_2B_ receptor. Even though the newly designed compound was not comparable in potency with the most potent existing antagonists, this study still provides an excellent application of *de novo* drug design in GPCR drug discovery field.

#### Cheminformatics and chemical genomics

While the number of currently available GPCR structures is increasing, it only covers a small portion of this protein superfamily and several other pharmaceutically relevant members are not yet elucidated. Chemical genomics can be applied to overcome the difficulty of target and drug identification by screening small molecule libraries and measuring their effects on entire biological systems or a specific group of targets, such as GPCRs. This combines the strength of traditional pharmaceutical techniques and genomics to facilitate discovery and validation of therapeutic targets, as well as identification of potential drug candidates for optimization (Hauser et al., 2018). Moreover, application of this strategy provides information concerning activated signaling pathways and biological effects through measurable gene expressions, leading to relevant data about target specificity and noninteraction pairs. In this sense, chemical genomics works on mining huge chemical data with the help of structural bioinformatics to rapidly identify target structure-function relationships (Valerio and Choudhuri, 2012). One of the most popular chemical genomics-based database found online is GLIDA (GPCR-Ligand Database), a publicly available Chemical Genomics database that can be used for GPCR drug discovery (Okuno et al., 2008). It contains GPCR biological and ligand information, as well as GPCR-ligand binding data. Therefore, it can be utilized for LBDD with the help of techniques such as machine learning-based classification and similarity-based search.

Shiraishi et al. reported an interesting research wherein chemical genomics approach was employed to predict GPCR-ligand interaction for class A GPCRs (Shiraishi et al., 2013). GPCR-ligand interaction data was collected from GVK Biosciences database and kernel methods were applied to evaluate compound-protein interaction (CPI) pair similarities based on Extended Connectivity Fingerprint (ECFP) and Dragon software descriptors generated for the ligands, along with target specific regions, such as full structure, loop region, and TM region. The results showed that compared to kernels accounting for the full structure and loop regions, kernels for the TM region showed significantly improved performance, which agrees with experimental findings that the TM region of class A GPCRs plays a critical role in ligand binding. Reliability of the machine learning model was improved with the addition of negative noninteraction pairs. Careful investigation of GPCR-ligand pairs revealed that high co-occurrence of residue-fragment pairs may be indicative of importance in ligand binding and specificity, as well as conservation of binding modes among Class A GPCRs. Key interactions identified in their study can be used for future VS and lead optimization studies and is beneficial when employed in combination with structure-based studies.

#### Cheminformatics, polypharmacology, drug repositioning, and repurposing

Recently, pharmaceutical research focuses not only on the discovery of novel compounds for a known target but also on the discovery of new indications for currently approved drugs. Polypharmacology has quickly emerged as a critical part of drug discovery research with the knowledge of how interconnected pathways in biological systems are. Though this field is most often used to investigate adverse effects and toxicity, information garnered from possible off-target effects can also offer information about new drug indications or cross-reactivity leading to higher drug efficacy (Jacobson et al., 2014). With the upsurge of polypharmacological information, it is no surprise that it is now frequently combined with cheminformatics strategies to predict off-target effects ahead of extensive biochemical analyses in order to save time and resources.

Xie et al. reported an interesting chemical genomics-based polypharmacology study focusing on GPCR-related drug abuse problem (Xie et al., 2014). Initially, a drug-abuse domain specific chemogenomics knowledgebase (DA-KB) was built to consolidate chemogenomics data regarding drug abuse and CNS diseases. This database was later used to investigate molecular interaction networks that encompass both drug abuse and GPCR modulation. Upon identifying 85 drug abuse-related GPCRs, distribution information of these receptors was collected and studied from the MetaCore database (Ekins et al., 2006). Using HTDocking (https://omictools.com/htdocking-tool) and GPCRDocking programs, polypharmacology and polydrug addiction analyses were performed to investigate the interactions between drug abuse-related receptors and ligands, along with cross-reactivities. As a result, the DA-KB became a powerful tool that has the capability of transforming data to useable polypharmacology knowledge. Moreover, TargetHunter server was also developed and can be used for target or off-target discovery.

### Cheminformatics approaches based on the GPCR structural data

SBDD is one of the potent tools in lead discovery and optimization (Andrews et al., 2014). The application of SBDD is proven to be more efficient than traditional methods due to its working principle, which includes understanding the molecular basis of the disease and utilizing the 3D structural data of the target protein in the drug discovery pipeline (Cavasotto and Palomba, 2015). It has played a valuable role in several drug discovery projects involving enzyme targets (Wlodawer and Vondrasek, 1998; Varghese, 1999). Due to the difficulties in the expression and crystallography of GPCRs, there was only limited information available for SBDD of such targets. However, methodological advances in GPCR crystallography have paved way for the elucidation of several GPCR structures in the recent past. The availability of GPCR structures led to increased application of structure-based approaches in GPCR drug design, an area which has long been dominated by ligand-based ones. Breakthroughs in GPCR structural biology provide invaluable insights into the GPCR structure, function, and polypharmacology. The abundance of ligand-bound GPCR structures unveils the intricacies of ligand-receptor interactions, thus triggering a shift from conventional HTS techniques to less cost and highly efficient SBDD approaches for the design and discovery of potent ligands with improved pharmacological profiles. The main drawback of SBDD approaches lies on the scoring functions used by docking algorithms, wherein numerous approximations and restraints to protein flexibility are applied to expedite the process (Kim and Cho, 2016). In the following section, we briefly discuss the structure-based cheminformatics approaches for identifying novel GPCR ligands targeting ligand- and/or allosteric binding sites with few thriving models from the literature.

#### Identification of GPCR novel chemotypes via structure-based virtual screening

**Figure 5:**
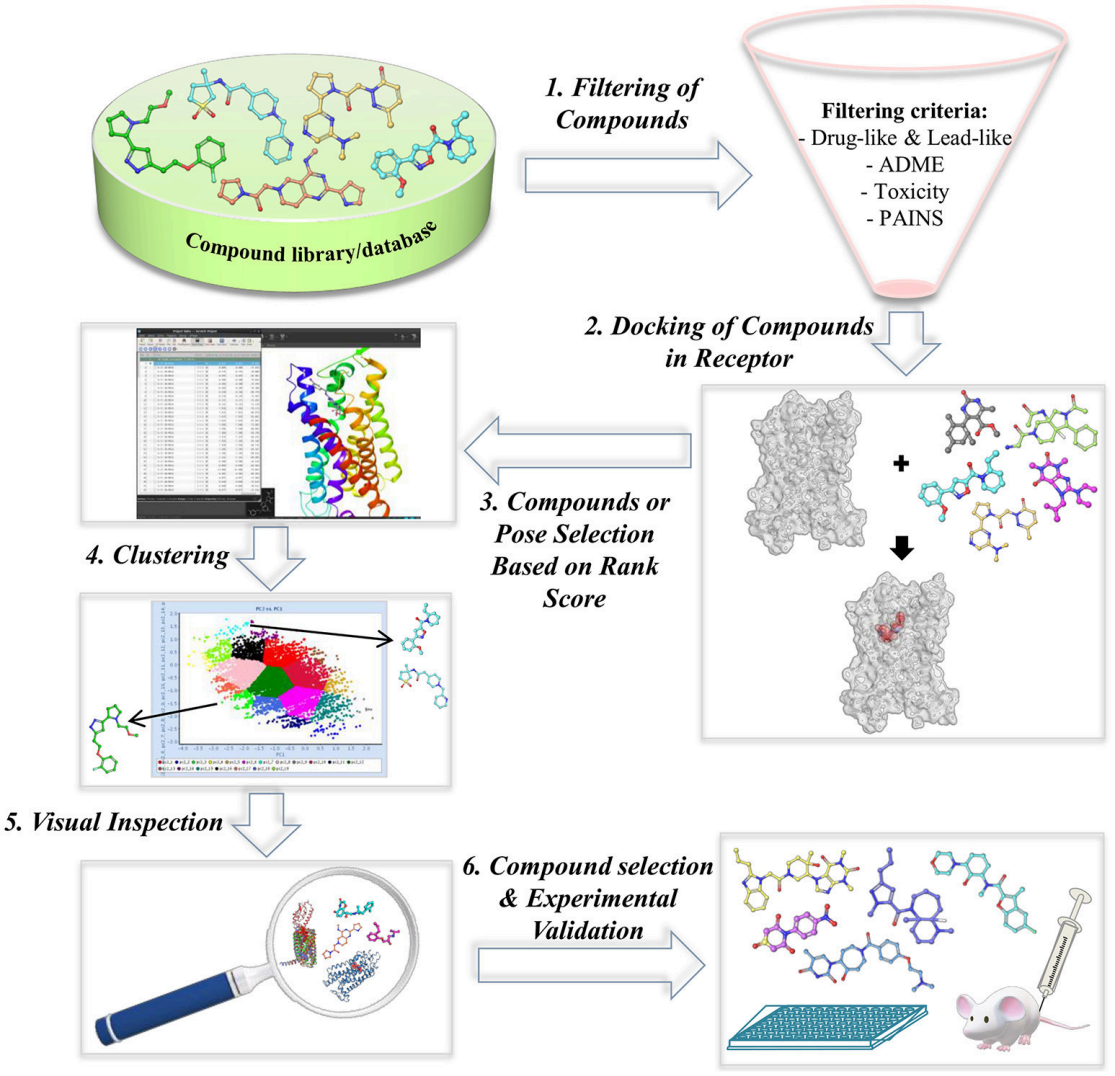
Overview of the typical workflow of structure-based virtual screening (SBVS).

Utilizing crystal structures or homology models of target proteins in rational drug design is considered as the most powerful and popular method of choice in the design and/or screening of new lead compounds. In the early phase of drug discovery pipeline, structure-based virtual screening (SBVS) or docking-based VS has been utilized for the prediction of novel bioactive compounds from large and chemically diverse libraries (Cheng et al., 2012). In general, SBVS requires knowledge about the target's (protein or receptor) 3D structural information determined through experimental (X-ray or NMR) or *in silico* methods (homology modeling). Procedure involves docking of large chemical libraries of small compounds into crystal structure or homology model of the receptor. The selection criteria of small compounds for further experimental testings are based on the docking score, which assesses the binding affinity of protein-ligand complexes, predicted binding poses, chemical diversity, interactions with key residues, etc. (Ngo et al., 2016). The small compounds that cause a biological response are known as hits, which act as new chemical scaffolds for hit-to-lead development. The general VS workflow applied in several GPCR VS studies is shown in Figure 5.

SBVS studies for the first crystal structures of GPCRs, including β_2_AR, A_2A_AR, dopamine D_3_R, and histamine (H_1_R) have shown high hit rates. The pioneering study of SBVS for a druggable GPCR using the β_2_AR crystal structure was reported (Cherezov et al., 2007). In another SBVS, the authors utilized the inactive structure of β_2_AR/carazolol (PDB code: 2RH1) (Sabio et al., 2008) and screened proprietary and public databases for the identification of β_2_AR ligands. The hit rates obtained were 36 and 12%, respectively. Similarly, Kolb et al. (2009) docked ~1 × 10^6^ commercially available compounds onto the same crystal structure and the top 25 virtual hits were selected based on their commercial availability, chemical diversity, and complementarity to the binding sites, and subjected for biological testings. Among them, six compounds had detectable binding affinities with the best one showing a Ki of 9 nM. All six hit molecules had novel chemotypes, and five of them were confirmed as inverse agonists. Apart from the reported VS studies using crystal structures, there were also few reports using receptor homology models. Langmead et al. identified highly potent and novel chemotype 1,3,5-triazine derivatives using A_2A_AR homology models (Langmead et al., 2012). A virtual library of 5.45 × 10^5^ compounds was screened and the initial hits were selected based on the shape geometry and electrostatic properties of the orthosteric site. A hit rate of 9% was obtained and the structures were modified and optimized using X-ray crystallography and structure-based optimization techniques. This series of optimization led to the successful identification of AZD4635 (HTL-1071), which is in phase 1 clinical trials for immunooncology (Jazayeri et al., 2017).

Interestingly, a large-scale VS study was carried out by Lane et al. (2013) for the identification of both orthosteric and allosteric ligands of D_3_R. Based on the crystal structure of D_3_R, two optimized D_3_R models were prepared. To account for protein flexibility, conformers of D_3_R models were generated and subsequently evaluated by VS performance, i.e., conformers that can separate D_3_R actives from decoys were selected for the following analyses. The Molsoft Screen Pub database, which contains 4.1 × 10^6^ compounds, was virtually screened using docking calculations. Top 300 hits in each model were selected and clustered by chemical similarity (0.3 Tanimoto distance). The top 25 compounds selected did not have a positively charged amine forming a conserved salt bridge to D110^3.32^, which is contrary to D_3_R apo model, but has interactions with TM1, 2, 3, and 7 as well as ECL1 and ECL2. These hits also reach dopamine and D110^3.32^ at the end of the orthosteric pocket. Finally, the predicted novel allosteric ligands were experimentally validated, showing distinct functional profiles on dopamine-signaling efficiency. Another SBVS approach identified nanomolar lead compounds for the melanin-concentrating hormone-1 receptor (MCH-1R) (Lionta et al., 2014). This approach combines GPCR molecular modeling, antagonist binding site prediction, design, synthesis, and a focused library screening. A primary hit compound from a pyranose-based VAST library was initially used for the construction of a high quality MCH-1R model. Furthermore, the model validation was performed using a virtual enrichment experiment, along with the model-driven structure-based expansion of the initial hit, for identification of potent interactions in the binding site. A SBVS of a library with ≤0.7 Tanimoto similarity to existing MCH-1R ligands provided a 14% hit rate and 10 unique chemotypes of potent MCH-1R inhibitors, including two nanomolar leads (Lionta et al., 2014).

*In silico* screening territory for classes B, C, and F largely remains uncharted due to the limited number of crystal structures available. Using SBVS approach, noncompetitive ligands (allosteric modulators) of related class B GPCRs, namely glucagon receptor (GLR) and glucagon-like peptide 1 receptor (GLP-1R), were identified (de Graaf et al., 2011b). Based on the crystal structure of corticotropin-releasing factor 1 receptor (CRF_1_R), a homology model for GLR was constructed. A database containing 1.9 × 10^6^ compounds was assessed for chemical similarity to the current GLR noncompetitive inhibitors and docked onto the TM cavity of GLR. Based on the protein-ligand interaction fingerprints (IFPs), 23 compounds were selected and subjected for *in vitro* evaluations. Only two compounds were found to dose-dependently inhibit the effect of glucagon. One hit that was predicted as inactive for GLR bound to GLP-1R and potentiated a response similar to the endogenous GLP-1 ligand. For class C GPCRs, successful *in silico* VS studies were carried out against the VFT crystal structures (orthosteric N-terminal domain) of metabotropic glutamate receptor subtypes, mGlu_3_R and mGlu_4_R (Selvam et al., 2010). Besides the above-mentioned studies of VS campaigns, there are several computational works reported in the literature to discover novel orthosteric ligands for various GPCRs (which is well summarized in several review articles; Andrews et al., 2014; Cavasotto and Palomba, 2015; Shonberg et al., 2015; Ngo et al., 2016; Lee et al., 2018). Since SBVS on GPCRs is too broad to cover in this section, we have summarized representative case studies reported in the last 5 years (2013–2017) in Table 1.

#### Relevance of fragment-based drug discovery (FBDD) on GPCR targets

Sequential piecing of fragments together to develop a novel lead compound is known as fragment-based drug discovery (FBDD) or fragment-based lead discovery (FBLD). FBDD is a potent scaffold-hopping and lead structure optimization tool for drug discovery projects and serves as an alternative to HTS (Matricon et al., 2017). The success of this approach in drug discovery campaign could be visualized by the increase in the number of compounds (originated from virtual fragment screens) entering clinical trials. A remarkable example of drugs identified *via* FBDD approach is vemurafenib, which was approved for the treatment of metastatic melanoma in 2011 (Baker, 2013). FBDD uses small molecules comprising ≤20 heavy atoms as a starting fragment for effective hit optimization. The main concept of this approach is to discover ligands that are smaller than a regular drug compound. The enlarged coverage of uncharted chemical space in fragment databases provides an exciting opportunity to find ligands after screening only a few thousand compounds (Chen et al., 2013). A fragment library can be designed and screened using molecular docking studies (Lee et al., 2018). The retrieved fragments could be further optimized using other computational approaches for growing, linking, or both.

Strategies utilized in the development of fragments into a lead compound include fragment growing, fragment linking, sequential docking, and group-based QSAR techniques. Fragment growth strategy initially begins with a fragment in the receptor' active site and allows extension of the fragment to maximize its interaction with the residues in the binding pocket. Fragment linking refers to the covalent linking of two or more fragments to form a single molecule which provides a new chemical scaffold in the active site. The application of FBDD to SBVS increases the structural space of hit-to-lead compounds. Even though ligands retrieved from fragment libraries lack selectivity and exhibit low affinity, they can be used as starting points for novel lead discovery. Despite its numerous advantages, there are still limitations associated with this approach, such as low accuracy prediction of fragment binding modes and rapid accumulation of errors. However, this approach proves to be useful when complemented with experimental techniques. Fragment screening of GPCR ligands *via* experimental methods (NMR, SPR, and X-ray crystallography) is challenging due to the difficulties in obtaining substantial amounts of functional protein, inherent conformational flexibility of the receptors outside the membrane, and low expression of the receptors (Lee et al., 2018). Therefore, *in silico* FBDD approaches could be utilized for GPCRs and other therapeutic targets. In the following paragraphs, we discuss the successful application of FBDD on GPCR drug discovery from literature.

The importance of *in silico* screening against GPCR protein structures or homology models to investigate novel fragment-like ligand chemical space is applicable for several GPCR targets. One of the first successful virtual fragment screening was developed by de Graaf et al. against doxepin bound human H_1_R crystal structure (de Graaf et al., 2011a; Shimamura et al., 2011). In this approach, molecular docking and receptor-ligand IFP protocols were combined to discover a chemically diverse set of new fragment-like H_1_R ligands. Out of 26 fragment-like compounds, 19 showed high binding affinity at the receptor level (hit rate 73%). Similarly, another structure-based virtual fragment screening (SBVFS) was performed against two GPCR targets, namely dopamine (D_3_R) crystal structure and H_4_R homology model structure, and an in-house fragment library of 12,905 fragments (Vass et al., 2014b). Additionally, molecular dynamics (MD) simulations were performed to represent different conformational states of the receptor orthosteric site (Vass et al., 2014b). Single structure- and ensemble docking screens were carried out for both receptors. The resulting 50 virtual hits were subjected for *in vitro* studies. Both the single and ensemble structures were found to be suitable for docking-based VS of fragments against GPCR targets. Chen et al. complemented *in silico* SBVFS with experimental biophysical screening to test the efficiency of their developed method (Chen et al., 2013). Initially, a set of 500 fragments were docked onto the orthosteric pocket of antagonist-bound A_2A_AR crystal structure (Jaakola et al., 2008) and ranked by affinity prior to target immobilized NMR screening of the same library (TINS). TINS resulted in 94 hits, where five fragments were identified to exceed the threshold affinity for the GPCR target. In the *in silico* screening, four out of five compounds were found in the top 50 fragments. Apart from these four fragments, the remaining 46 fragments also showed high binding affinities. Thus, a second computational screening approach using commercially available fragments (3.28 × 10^5^) was performed and the 22 top-ranked compounds were tested experimentally. Among them, 14 fragments were identified as A_2A_AR ligands. Furthermore, QSAR studies were performed for three potent A_2A_AR ligands followed by optimization of the fragments by MD simulations and free-energy calculations. Similarly, another successful application of fragment-based screening and lead optimization using both biophysical and *in silico* techniques was shown in β_1_AR target leading to the discovery of novel high affinity leads (Christopher et al., 2013).

Verheij et al. studied target selectivity against histamine subtype H_4_R and 5-HT_3A_ (ion channel) homology models using SBVFS approach (Verheij et al., 2011). The results of fragment-based screening showed that both receptors yielded a common pool of hit fragments, thus underlining remarkable similarities in ligand recognition. This knowledge could assist in efficiently navigating chemical space during hit optimization. Besides the orthosteric binding site (primary), allosteric sites (secondary) have also been targeted for identification of novel compounds by SBVFS approach. Vass et al. applied a sequential docking protocol to predict starting points for fragment linking using D_3_R crystal structure and D_2_R homology model to identify subtype selectivity (Vass et al., 2014a). Two in-house focused fragment libraries (196 fragments function as primary binding site ligands for D_2_ and D_3_ receptors and 266 fragments function as secondary binding site ligands for D_3_R) were docked in the orthosteric and allosteric binding sites and the best fragment combinations were listed. Similar top-scoring fragments were identified for the orthosteric site, whereas allosteric site fragments showed subtype selectivity. Three fragment-linked compounds that showed 9-, 39-, and 55-fold selectivity for D_3_R were synthesized, and docking results were validated by the experimental data.

In tandem with SBDD, FBDD has also been successfully applied to other GPCR classes. Novel mGlu_5_R NAMs were identified through combination of fragment-based screening and medicinal chemistry approaches (Christopher et al., 2015). In addition, the binding modes of NAMs with the receptor were crystallographically solved. Recently, an *in silico* fragment-based approach was applied on the crystal structures of mGlu_5_R (Doré et al., 2014; Christopher et al., 2015) for the design of novel allosteric modulators (Bian et al., 2017). Initially, a fragment library for reported GPCR allosteric modulators was constructed using the data from Allosteric Database (ASD). Subsequently, the novel compounds were generated and analyzed using retrosynthetic combinatorial analysis procedure (RECAP). Molecular docking was applied to screen the hits for the target by docking the *in silico* generated compounds into the binding pocket. Additionally, other computational methodologies, such as benchmark dataset verification, docking, QSAR model simulations, etc., were performed to assess validation of the hits. Twenty structurally diverse hits were predicted as potential mGlu_5_ allosteric modulators based on the binding energies and docking scores. This study highlights the importance of purely computational FBDD approach for facilitating the design of novel compounds for other targets as well. In addition to the above-mentioned GPCR case studies on SBVFS campaigns, there are several other *in silico* reports available regarding the discovery of novel ligands which are summarized elsewhere (Hubbard and Murray, 2011; Murray et al., 2012; Shoichet and Kobilka, 2012; Visegrády and Keseru, 2013; Andrews et al., 2014; Lee et al., 2018).

### Integration of ligand- and structure-based cheminformatics approaches

The use of cheminformatics in drug discovery provides an excellent foundation for the integration of structure- and ligand-based strategies due to its application in different stages of drug discovery. With the rising number of available structures, biological databases, and *in silico* techniques for cheminformatics and modern drug discovery, it is not surprising that ligand- and structure-based approaches are used in combination to take advantage of the abundant GPCR ligand information while employing recently elucidated crucial protein structural information to aid in increasing success in GPCR drug discovery research. Furthermore, integration of LBDD and SBDD complements strengths and weaknesses of each method, leading to better insights in critical ligand functionalities and receptor-ligand interaction information. Researchers are now able to use 3D protein structures to predict binding modes and study the pharmacology of known drugs and their analogs through docking, providing rationalization of ligand activity and useful SAR information for the design and optimization of new agonists and antagonists (Munk et al., 2016). In addition, rapid innovation of hardware and computing power allows the use of MD simulations for more in-depth study of GPCR ligand binding and activity modulation (McRobb et al., 2016; Clark, 2017).

An excellent case of ligand- and structure-based integration in GPCR drug discovery is shown in studies involving A_2A_AR, an attractive drug target for the treatment of Parkinson's disease. Since A_2A_AR receptor was one among the first GPCRs to be crystallized, it has become one of the most extensively studied drug target. The later release of a high-resolution A_2A_AR structure, which revealed the presence of water in the binding site, further increased the efforts for drug design and optimization. Over the years, most of A_2A_AR antagonists, such as istradefylline (Jenner, 2005) and preladenant (Neustadt et al., 2007), have been designed based on the purine scaffold and other related heterocycles. Although the abundance in ligand information for A_2A_AR helps in the elucidation of important chemical fingerprints and ligand binding interactions, it has become difficult to discover novel entities for drug development. In a study by Lenselink et al. (2016a), they performed VS using an ensemble of A_2A_ receptor structures split into a structure-based decision tree (Lenselink et al., 2014). Ligands were docked to each protein structure and proceeded to the next receptor docking based on a GlideScore cut-off of the previous procedure. The resulting ligands were filtered using Rapid Elimination of Swill (REOS) (Walters and Namchuk, 2003) and re-scored using MM-GBSA. Consequently, similarity-based analysis (against compounds tested for A_2A_AR activity recorded in ChEMBL) was performed to determine the structural novelty of the remaining hits and select the most unique compounds to be tested experimentally. Out of 71 novel ligands, only 2 compounds displayed suitable A_2A_AR binding affinity. They also performed a retrospective analysis of the current A_2A_AR ligands to determine novelty in structure and its relation to observed A_2A_AR activity. Decades of research efforts for this target left little room for discovery of new ligand scaffolds, as seen in previous VS studies showing ligand Tanimoto similarity in the range of 0.19–0.68 (Carlsson et al., 2010; Katritch et al., 2010; Langmead et al., 2012; Rodriguez et al., 2015), with the lowest similarity showing the least activity. While most of the virtual hits were found to be similar in structure to experimentally validated compounds from ChEMBL, it should be noted that several of the tested compounds or scaffold structures were also discovered using computational methods, highlighting the value of *in silico* approaches in drug discovery and design.

Aside from combining known structure- and ligand-based methods, hybrid tools that assimilate features from both approaches have been developed to afford computational chemists other strategies which can compensate current individual limitations of SBDD and LBDD. One of the hybrid methods that has gained popularity in recent years is proteochemometric (PCM) modeling. PCM modeling is similar to traditional QSAR studies since both methods require descriptors, bioactivity data, and machine learning functions for model development (Qiu et al., 2017). However, a cross-term descriptor is also required in PCM modeling to consider amino acids and ligand functional groups that are crucial for binding interaction of the complex (Lapinsh et al., 2001; van Westen et al., 2011; Qiu et al., 2017). This method has been found to be useful on polypharmacological studies as it can provide information on target selectivity (Cortes-Ciriano et al., 2015), especially in large protein families like GPCRs. In a recent study by Gao et al. (2013), 24 PCM models were developed for amine GPCRs and their corresponding ligands using machine learning methods, support vector regression (SVR), and Gaussian processes (GP). Two typical descriptors were generated per receptor: z-scale and transmembrane identity descriptors, and two typical descriptors were generated for each ligand: general (atomic contributions, logP, etc.,) and drug-like index descriptors. These descriptors were first used to build 24 PCM models, which were validated using a test-set. Although, most of the models showed strong goodness-of-fit (R^2^) and predictivity (Q^2^), the addition of cross-terms led to a lower predictive capability of the PCM models. This may be because it is still difficult to fully translate receptor-ligand interfaces to a descriptor value. Despite this, their PCM models showed great potential in predicting cross interactions between GPCRs and ligands.

## Summary of cheminformatics softwares/tools utilized in GPCR drug discovery

HTS has undergone technological advances and innovations that has rendered it as the principal method of drug discovery for years. However, it did not necessarily lead to a great leap forward in the discovery of NCEs as the hit rate for this method is frequently low, in addition to the enormous costs and efforts involved. In turn, computer-aided drug design (CADD) have been recognized and continuously receives increase in interest and usage such that most of GPCR drug discovery research efforts make use of one or more computational tools, especially in the initial stages of drug design. Due to the complexities of experimental GPCR research, it is of no surprise that CADD has emerged as a method of choice to expedite GPCR drug discovery and design. Furthermore, increasing knowledge of GPCR systems has led to the rising popularity of cheminformatics and chemogenomics as evidenced by the growing number of publicly available databases, which can provide structural or interaction information regarding receptor and its associated ligands.

There are several cheminformatics softwares and web servers available to identify lead compounds targeting GPCRs (Khan et al., 2011; Yadav et al., 2016). As mentioned previously, *in silico* approaches are classified into two approaches: SBDD and LBDD. If there are already known NMR and X-ray crystal structures or reliable homology models available, computational methods based on target protein structures can be exploited (Lyne, 2002). These tools are related with several computational approaches, including molecular docking, VS, pharmacophore generation, and binding pocket detection. As shown in Table 3, several *in silico* cheminformatics methods have been applied for GPCR targeted drug discovery. In cases where no protein structures are available, ligand-based virtual screening (LBVS) can be utilized. LBVS can be further sub-classified into three: pharmacophore-, similarity-, and machine learning-based VS (Basith et al., 2016). As shown in Table 4, several *in silico* cheminformatics methods could be exploited for generation of pharmacophores, searching 3D similarity, and identifying targets (polypharmacology). Moreover, commercially available chemical libraries for VS are shown in Table 5.

**Table 3:** Cheminformatics tools for structure-based drug discovery.

**Tools**	**Description**				**Availability**	**References**
	**VS**	**Docking**	**Pharmacophore generation**	**Cavity detection**		
AutoDock 4	Y	Y			Public	Morris et al., 2009
AutoDock Vina	Y	Y			Public	Trott and Olson, 2010
FlexX	Y	Y			Commercial	Kramer et al., 1999
OEDocking (FRED, HYBRID)	Y	Y			Commercial	McGann, 2012
Galaxy7TM		Y			Public	Lee and Seok, 2016
Glide (HTVS, SP, XP)	Y	Y			Commercial	Friesner et al., 2006
GOLD	Y	Y			Commercial	Jones et al., 1997
GOMoDo	Y	Y		Y	Public	Sandal et al., 2013
GPCR automodel		Y			Public	Launay et al., 2012
ICM-Pro	Y	Y			Commercial	Neves et al., 2012
MOE	Y	Y		Y	Commercial	Roy and Luck, 2007
Snooker	Y		Y			Sanders et al., 2011
Surflex-Dock		Y			Commercial	Jain, 2007
fPocket	Y			Y	Public	Le Guilloux et al., 2009
Pocketome				Y	Public	Kufareva et al., 2012
UCSF DOCK		Y	Y		Commercial	http://dock.compbio.ucsf.edu/
MOLS		Y			Public	Paul and Gautham, 2016
iScreen	Y	Y		Y	Public	Tsai et al., 2011

**Table 4:** Cheminformatics tools for ligand-based drug discovery.

**Tools**	**Description**				**Availability**	**References**
	**VS**	**Pharmacophore generation**	**3D similarity searching**	**Poly pharmacology**		
Discovery studio	Y	Y			Commercial	
FlexS			Y		Commercial	Lemmen et al., 1998
ICM-Pro	Y	Y	Y	Y	Commercial	Grigoryan et al., 2010
LigandScout	Y	Y			Commercial	Wolber and Langer, 2005
PharmaGist	Y	Y			Commercial	Schneidman-Duhovny et al., 2008
QSARPro	Y		Y		Commercial	http://www.vlifesciences.com
ROCS			Y		Commercial	Hawkins et al., 2007
Surflex-Sim			Y		Commercial	Spitzer and Jain, 2012
Swiss similarity			Y		Public	Zoete et al., 2016
Topomer CoMFA	Y		Y		Commercial	Cramer, 2003

**Table 5:** Available chemical database for high-throughput virtual screening.

**Database**	**Number of compounds**	**Containing GPCR focused library**	**References**
AnalytiCon	35,000		https://ac-discovery.com
Asinex	600,000	Y	http://www.asinex.com
Bionet	80,700		https://www.keyorganics.net
ChemBridge	1,100,000	Y	http://www.chembridge.com
ChemDiv	1,500,000	Y	http://www.chemdiv.com
CoCoCo	6,981,500		http://cococo.isof.cnr.it/cococo
eMolecules	5,900,000		https://www.emolecules.com
Enamine	2,300,000	Y	http://www.enamine.net
InterBioScreen	550,000		https://www.ibscreen.com
Life Chemicals	1,292,000	Y	http://www.lifechemicals.com/
Maybridge	53,000		http://www.maybridge.com/
NCI	260,000		https://cactus.nci.nih.gov/
OCTVAchemicals	260,000	Y	http://www.otavachemicals.com
Prestwick Chemical	1,280		http://www.prestwickchemical.com
Selleck Chemicals	482	Y	http://www.selleckchem.com
SuperDrug2	3,900		http://cheminfo.charite.de/superdrug2/downloads.html
TCM Database	32,300		http://tcm.cmu.edu.tw/
Timtec	2,300	Y	http://www.timtec.net/
Vitas-M	1,500,000		http://www.vitasmlab.com
ZINC	35,000,000		Irwin et al., 2012

## Limitations of cheminformatics approaches in GPCR drug discovery

In the last several years, the increasing number of high resolution GPCR structures has unlocked new avenues for structure-based GPCR drug discovery and design. However, several obstacles remain, including rapid identification of novel fragment-like compounds and structure-based elucidation of GPCR ligand function to name a few.

With the recent innovations in high-throughput, computer, and software technologies, as well as the upsurge of publicly available data, cheminformatics methodologies has no doubt become an essential part of most drug discovery efforts to date. However, a major flaw is seen during cheminformatics model development, wherein the experimental data used is assumed to be correct. In contrast to this assumption, databases can contain errors for ligand structures, bioactivity, activity types, and other information, which often results in ambiguous models leading to erroneous findings. Several recent articles (Fourches et al., 2010, 2016; Williams and Ekins, 2011; Williams et al., 2012) have discussed this topic at length and how it can have a negative effect on model development and performance. A study by Olah et al. (2005) mentioned that there were two molecules with incorrect structures on average for each medicinal chemistry journal, indicating a total error percentage of 8% in the WOMBAT database. Another more recent study by Tiikkainen et al. (2013), estimated the ligand error rates in ChEMBL, Liceptor, and WOMBAT databases to be 5, 7, and 6%, respectively. Error values for activity values in the three databases ranged from 1 to 2%. It is therefore important to carefully and manually curate chemical and biological databases, since even minor errors can cause a substantial decrease in the predictive capability of generated models. Moreover, while the increasing sophistication of computer programs has allowed researchers an atomistic view of several GPCR systems, approximations of crucial energy terms that cannot be computationally explored at present has greatly limited the accuracy in the perception of these systems. Because of these, researchers should constantly gauge findings against their own scientific knowledge to see whether the results are significant or not. It should always be remembered that computational tools are created and continuously developed to assist in making the drug discovery process more efficient, but nothing can replace a researcher's own knowledge and experience.

Moreover, insights about GPCR structure, function, and binding partners have increased significantly compared to a few decades ago. Despite this, a great deal of information is still beyond our fingertips, such as protein structures of hundreds of unique GPCRs and ligand information for orphan GPCRs. It is imperative not lose fervor in gathering new knowledge to further enhance our understanding of GPCR structures and functions.

## Conclusions

In the nineteenth century, chemical space exploration was initiated as a counting game to estimate its size (Reymond, 2015). However, the advent of cheminformatics field and powerful *in silico* technologies assisted in the exploration of uncharted ligand space from large chemical libraries. The availability of large public and commercial chemical databases, as well as ligand chemical space exploration tools, provide researchers the ease of accessibility to handle and explore huge chemical data. Cheminformatics is a complex field of study that translates large data into useful knowledge for drug design and optimization protocols. The expansion of GPCR structures and ligands over the past decade is mainly due to the progress in its structural biology and theoretical advancements. These structural and *in silico* breakthroughs have led to the implementation of cheminformatics approaches in GPCR drug discovery pipeline. In the GPCR drug discovery protocol, ligand- and structure-based approaches are the most commonly applied ones. LBDD is known as a fast and simple technique for the identification of vital chemical functionalities required for biological activity. However, absence of binding pocket information limits its ability in incorporating several important factors, such as receptor flexibility and ligand bioactive conformation, thereby restricting the discovery of candidate leads to only the ligand classes used in model development (Saxena et al., 2017). But due to the prolonged absence of GPCR structures, researchers relied heavily on ligand-based methods for drug discovery and lead optimization, leading to copious ligand structural information for these targets. Following the crystallization of bRho in 2000 (Palczewski et al., 2000) and β_2_AR in 2007 (Rasmussen et al., 2007), a striking increase in GPCR structural information have been observed in the last several years. While the current available structures are unable to cover the structural diversity of GPCR protein family members, there is enough that can be used as templates for homology modeling to perform SBDD. In contrast to ligand-based techniques, SBDD can be used to predict ligand bioactive conformation, thus providing a better understanding of receptor-ligand interactions and allowing the discovery of NCEs. Furthermore, recent researches underpin the significance of emerging integrated approaches in GPCR drug design and discovery. Assimilating LBDD and SBDD methods, as well as the use of integrated approaches, has proven to increase the success rate of finding promising leads, especially for well-studied targets such as GPCRs. All the cheminformatics approaches discussed in this review are focused toward the identification of novel ligands for GPCR targets based on the structural and ligand data, where several case studies signify the importance of VS. The evolution of cheminformatics techniques and their synergy in GPCR drug discovery pipeline is the driving force that will facilitate cost-effective and prolific outcomes in the exploration of uncharted GPCR ligand space. Yet, an expert human touch is entailed to authenticate and tame the computer-generated outcome.

## Author contributions

SB and MC summarized the literature, wrote the manuscript, and prepared the figures. SM wrote part of the manuscript, prepared the figures, and revised the manuscript. JP and NC prepared the tables. SK and SC supervised all the works, provided critical comments, and wrote the manuscript.

### Conflict of interest statement

The authors declare that the research was conducted in the absence of any commercial or financial relationships that could be construed as a potential conflict of interest.
